# Changing Selective Pressure during Antigenic Changes in Human Influenza H3

**DOI:** 10.1371/journal.ppat.1000058

**Published:** 2008-05-02

**Authors:** Benjamin P. Blackburne, Alan J. Hay, Richard A. Goldstein

**Affiliations:** 1 Division of Mathematical Biology, National Institute of Medical Research, Mill Hill, London, United Kingdom; 2 Division of Virology, National Institute of Medical Research, Mill Hill, London, United Kingdom; Emory University, United States of America

## Abstract

The rapid evolution of influenza viruses presents difficulties in maintaining the optimal efficiency of vaccines. Amino acid substitutions result in antigenic drift, a process whereby antisera raised in response to one virus have reduced effectiveness against future viruses. Interestingly, while amino acid substitutions occur at a relatively constant rate, the antigenic properties of H3 move in a discontinuous, step-wise manner. It is not clear why this punctuated evolution occurs, whether this represents simply the fact that some substitutions affect these properties more than others, or if this is indicative of a changing relationship between the virus and the host. In addition, the role of changing glycosylation of the haemagglutinin in these shifts in antigenic properties is unknown. We analysed the antigenic drift of HA1 from human influenza H3 using a model of sequence change that allows for variation in selective pressure at different locations in the sequence, as well as at different parts of the phylogenetic tree. We detect significant changes in selective pressure that occur preferentially during major changes in antigenic properties. Despite the large increase in glycosylation during the past 40 years, changes in glycosylation did not correlate either with changes in antigenic properties or with significantly more rapid changes in selective pressure. The locations that undergo changes in selective pressure are largely in places undergoing adaptive evolution, in antigenic locations, and in locations or near locations undergoing substitutions that characterise the change in antigenicity of the virus. Our results suggest that the relationship of the virus to the host changes with time, with the shifts in antigenic properties representing changes in this relationship. This suggests that the virus and host immune system are evolving different methods to counter each other. While we are able to characterise the rapid increase in glycosylation of the haemagglutinin during time in human influenza H3, an increase not present in influenza in birds, this increase seems unrelated to the observed changes in antigenic properties.

## Author Summary

H3N2-type influenza is responsible for widespread disease and significant mortality. The virus evolves rapidly, changing its antigenic properties, allowing it to escape clearance by the immune response as well as complicating the maintenance of vaccine effectiveness. Part of this evolution has been the rapid increase in glycosylation, an increase not observed either in H9 evolution in birds or in H1 evolution in humans. It has been observed that the antigenic properties change in a punctuated, discontinuous manner. This could be either because some mutations are more significant than others, or it could mean that the antigenic changes correspond to adjustments in the antagonistic relationship between virus and host. By studying the sequence evolution of the H3 haemagglutinin, we can demonstrate that the selective pressure acting on the virus protein changes with time, and that these changes are especially rapid during changes in antigenic properties. This indicates that the antigenic changes correspond to modifications in the virus–host relationship. Surprisingly, neither the changes in selective pressure nor the changes in antigenic properties correspond to changes in glycosylation.

## Introduction

The rapid evolution of influenza viruses presents difficulties in recognising and predicting current and future epidemiological threats. One of the major sources of information about possible future threats from influenza is a study of its history as an evolving pathogen. Analysing how the virus evolves to evade the immune response can provide insight into how the immune system has dealt with the virus in the past and how the virus may change in the future to evade elimination.

Modelling of influenza evolution has focused on haemagglutinin (HA), the membrane-bound glycoprotein present on the surface of the virus which is responsible for receptor-binding and membrane-fusion. Sixteen different HA subtypes in influenza A have been identified (H1 to H16) of which H1 and H3 are currently circulating in human populations. For membrane fusion to occur, the HA precursor (HA_0_) must be cleaved into two polypeptides, HA_1_ and HA_2_, linked by a disulphide bond. Five canonical antigenic sites have been identified on the HA_1_ polypeptide of H3 [1],[2]. Because HA_1_ is the principal target of antibody-mediated immunity [3], it has a higher replacement rate for amino acids than HA_2_ [4].

The sequence evolution of HA_1_ results in *antigenic drift* as antigenic properties change with time. Amino acid substitutions result in changes in the ability of antibodies to neutralise the virus, either through interfering with antibody binding or changing some associated property (*e.g.* receptor binding), so that antisera raised in response to one virus have reduced effectiveness against a future virus [4]. The amount of this reduction can be used as a measure of the difference between the antigenic properties of the two viruses. Interestingly, antigenic properties of H3 move in a discontinuous, step-wise manner [5]. For periods of two to five years, HA_1_ sequence evolution has a limited effect on virus-antibody interactions, so that antigenic drift is confined to a semi-well-defined group of sequence variants with similar antigenic properties, what has been referred to as an *antigenic cluster*. Periodically, however, sequence change results in significant change in antigenic properties, corresponding to a jump to a new antigenic cluster. The correlation between genetic distance and antigenic distance from the root cannot be explained by a linear relationship [5].

There are two possible, not mutually-exclusive interpretations of these irregular, punctuated changes. Firstly, it would be expected that changes in different locations would have different impacts on the antigenic properties, what has been described as the ‘influential sites’ model of antigenic change [6]. Some changes in the sequence will cause insignificant changes in antigenic properties, while other changes, near the antigenic or binding sites, would be more important. A constant rate of change of the sequence would result in punctuated changes in antigenic properties if relatively few locations had a very large effect on these properties [3]. (In the data analysed by Smith and co-workers, jumps between antigenic clusters can result from a single amino-acid substitution [5].)

Secondly, it might be that each antigenic cluster represents a particular manner of interaction between the virus and the host, such as nature or location of antibody binding. Changes in the amino acids at some locations would cause jumps between antigenic clusters, representing changes in this relationship. As a result, the effect of subsequent amino acid changes at other locations might be significantly different. In particular, Koelle et al. recently performed a simulation of the effect of such context-dependent interactions on the evolutionary dynamics of influenza, showing that it could re-create many observed epidemiological patterns [6].

One way to distinguish between these two possibilities is to look at the changing patterns of *selective pressure*. If changes in the antigenic cluster correspond to modifications in virus-host interactions, we would expect there to be corresponding changes in the selective pressure at different locations in the viral proteins. As the relative and absolute rates of amino acid substitution at these locations will depend upon the nature of the local selective pressure, we might be able to observe a change in the pattern of amino acid substitutions. These changes can be in the overall rate of substitution as well as in the nature of the substitutions accepted.

One possible cause of these punctuated antigenic changes is the changing glycosylation state of the haemagglutinin. There has been a large increase in the number of predicted HA_1_ glycosylation sites from viruses isolated in 1968 to those circulating at present [3],[7]. It would seem possible that the addition of these glycosylation sites represent a way for the virus to avoid the immune response through gross changes in the protein exterior. If these changes in glycosylation are related to changes in antigenic properties, we might expect correlations between the changes in glycosylation, changes in antigenic properties, and changes in the selective pressure at various locations in the protein.

In addition to constructing phylogenetic trees, evolutionary theory can also be applied to a wide range of problems through hypothesis testing. These approaches can generate new insights into the forces of evolution that are shaping the protein sequence, and hence into the structure, function, and physiological context of the protein itself [8]. Competing models of sequence change can be applied to the data, and standard tools from statistics and information theory can be used to evaluate the evidence for specific behaviour. In this paper we are interested in addressing specific questions regarding haemagglutinin evolution. Does the selective pressure change during evolution, either in degree or nature? Are the changes in selective pressure correlated with changes in antigenic properties or changes in glycosylation?

We develop a series of increasingly-complex models for the evolution of HA_1_ of human H3N2 viruses, at each stage inquiring whether we have statistical grounds for rejecting the simpler model. We start with a standard model where the rate of amino acid change at all locations is modelled by a single substitution matrix, allowing for heterogeneity of overall substitution rate following a Gamma distribution. We then develop a so-called ‘mixture model’ where different locations in the protein follow one of a set of possible substitution matrices, differing in overall substitution rate as well as different propensities for the various amino acids, but where we assume the substitution rates at any location is constant over the evolutionary process. The next model allows changes in the substitution rates during the evolution, corresponding to changing selective pressure. Because our mixture model includes substitution matrices that differ in their preference for different amino acids, we can detect changes in the nature of the selective pressure that do not correspond to changes in the magnitude or sign. Finally, we consider a more complicated model that considers that the alterations in selective pressure might preferentially occur on branches of the evolutionary tree corresponding either to changes in antigenic cluster or to changes in glycosylation.

We find different substitution matrices describing different regions of the protein, indicating a range of selective pressures. We also find that these selective pressures change with time. More specifically, changes in selective pressure do not seem to occur at a constant rate throughout the tree. Rather, changes in selective pressure are found to occur more often during major changes in antigenic properties. This suggests that the movement between the antigenic clusters, as observed in Smith *et al.* [5] corresponds to changes in the nature of the interaction between virus and host. The locations that undergo changes in selective pressure are largely in places under positive selection, at or around the cluster-difference substitutions (identified by Smith *et al.*), and in locations in canonical antigenic sites. Surprisingly, we do not observe a significant correlation between rapid changes in antigenic properties and changes in the predicted HA glycosylation state. Nor do we observe changes in glycosylation state during rapid changes in antigenic properties. This indicates that changes in glycosylation do not play a dominant role in the major changes of antigenic properties.

## Results

A phylogenetic tree was constructed for the sequences used in the analysis of Smith *et al.* [5], as drawn schematically in Figure S1. (The detailed tree is available from the authors.) The various clusters of sequences with similar antigenic properties is notated by the location (Hong Kong, ENgland, VIctoria, TeXas, BangKok, SIngapore, BEijing, WUhan, SYdney, FUjian) and year of the earliest sequence. The derived tree is similar to that derived by Smith *et al.* [5]. In contrast to their tree, however, we have transitions from antigenic clusters EN72 to VI75 and TX77, with VI75 representing a dead-end, as well as transitions from SI87 to both BE89 and BE92, with BE89 representing a dead-end.

We then applied a series of different evolutionary models to these data, using various tests to quantify the statistical support for the increased complexity. As described in the Methods section below, if the models are nested (that is, the simpler model is a special case of the more complex model), we can use the likelihood-ratio test to determine the statistical support for rejecting the simpler model. When the models are not nested, we use the Akaike information criterion (AIC), which penalises more complex models based on the number of adjustable parameters; the best model is the one that minimises the AIC.

### Choice of model and evaluation of model parameters

We developed four different evolutionary models. Model 1, representing a standard optimised single substitution-model with Gamma-distributed rates, when applied to the haemagglutinin sequence data, yielded a log-likelihood of −4674.1, for AIC = 10,492.2. (The number of parameters includes 209 model parameters and 363 adjustable branch lengths.) We then developed a mixture-model (Model 2) where there were a number of different substitution matrices representing different forms of selective pressure, defined by an overall substitution rate and different relative propensities for the various amino acids. Model 2 assumed that the selective pressure acting on every location was constant with time throughout the evolutionary process. The number of substitution matrices was optimised by minimising the AIC. The best performance was obtained with a mixture-model with four substitution matrices (271 adjustable model parameters), which achieved a log-likelihood value of –4339.2 for a substantially lower AIC = 9,946.4. This indicates that an evolutionary model including qualitatively-different forms of selective pressure at different locations fits the data significantly better than a single substitution matrix with a Gamma distribution of rates.

Allowing changes of selective pressure during the evolutionary process (Model 3) increased the log-likelihood to −4319.8. Model 2 (no changes of selective pressure) is nested in Model 3, meaning we can use the likelihood ratio test to demonstrate that the extra parameters can be justified (P<10^−4^). We then tried a more elaborate model (Model 4), where the branches that involved changes of antigenic cluster had a greater amount of change of selective pressure. This increase of one additional adjustable parameter resulted in an increase in the log-likelihood to −4311.8, indicating that this extra complexity is justified (P<10^−4^ with the likelihood-ratio test), and that the increased rate of selective-pressure change between antigenic clusters is statistically-significant. We can then reject the null hypothesis that changes in selective pressure occur independently of jumps in antigenic properties, in favour of a model where changes in selective pressure occur preferentially coincident with such jumps. The rate of substitution-matrix change for the inter-cluster branches was γ = 0.77; that is, the extra amount of substitution matrix (selective pressure) changes is equivalent to what would be observed if the branch lengths corresponding to antigenic cluster transitions were increased by this amount.

### Changes in glycosylation

We performed ancestral reconstruction, and predicted glycosylation states of the various ancestral nodes. We restricted our analysis of glycosylation sites to the sites predicted on the ancestral nodes with probability >0.95. We did not consider changes in glycosylation of the terminal sequences, as these might represent deleterious mutations, cannot be associated with changes in selective pressure with our model (as there is no sequence evolution observed following the terminal sequence), and are independent of changes in antigenic cluster.

**Figure 1:**
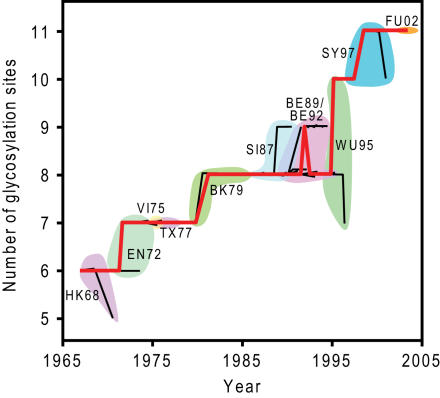
Changes in the number of glycosylation sites with time. Clusters are indicated as block colouring. Thick red line represents the main branch of the tree. Dates for the internal nodes were estimated based on extrapolating a linear least-squares fit of the acquisition time of the available sequences.

Predicted glycosylation states are tabulated in Table S1. We observe a sharp increase in the amount of predicted glycosylation sites from 6 sites (HK68) to 11 (FU02), as shown in Figure 1. Interestingly, there seems to be no correlation between changes in glycosylation and major changes in antigenic properties, with *no* transition between antigenic clusters corresponding to a difference in glycosylation. Conversely, some antigenic clusters contain a multiplicity of different glycosylation sites; WU95 viruses, for instance, contain between 7 and 10 glycosylation sites per subunit. This suggests that the rapid change of glycosylation is disjoint from the major changes in antigenic properties.

We identified branches corresponding to a change in glycosylation. We then developed a substitution model (Model 5) where the branches involving changes in glycosylation state were affected by an additional amount of selective-pressure change. There was a minimal change in log likelihood, indicating that there was not a significant observed correspondence between changes in glycosylation and changes in selective pressure, given the available data (P = 0.8). We find no evidence that changes in glycosylation correspond to significantly increased probability of changes in selective pressure.

### Evaluation of model

The results described in more detail below refer to Model 4, unless specified otherwise.

**Figure 2:**
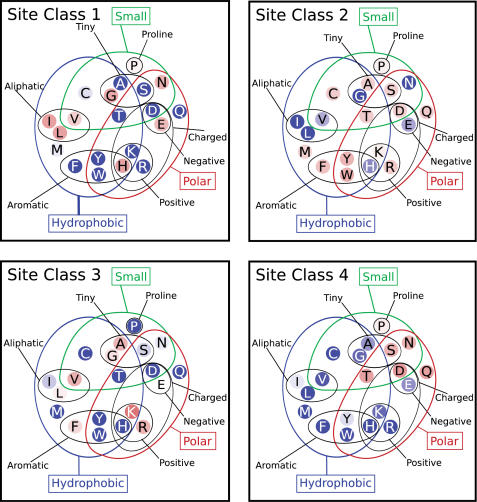
Characteristics of the four substitution matrices. Each substitution matrix is represented by the Venn diagram of physical properties devised by Taylor [43]. Non disulfide-bonded cysteines have been excluded from the figure, as all cysteines in HA1 are disulfide bridged. Each substitution matrix is characterized by a relative overall substitution rate and different propensities for the various amino acids represented by equilibrium frequencies. Amino acids in this figure are colored according to these equilibrium frequencies compared with the overall average. Blue indicates a frequency is less than the mean, with red amino acids greater. More intense colors are proportionally further from the mean [44].

**Figure 3:**
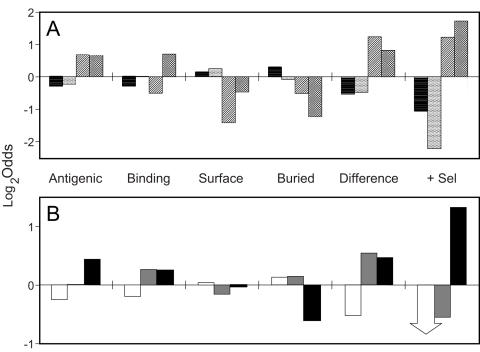
A) Log2-odds representation of the propensity of various types of locations for various substitution matrices in HA1, indicating the log2 of the relative frequency of a given substitution matrix in each of the types of locations divided by what would be expected at random. Substitution-matrix assignments are averaged over all of the internal nodes of the phylogenetic tree. Eight different types of location types are considered: antigenic (‘Antigenic’), receptor binding sites (‘Binding’), non-antigenic non-binding exposed sites (‘Surface’), buried sites (‘Buried’), sites of ‘cluster-difference’ substitutions (Smith et al. 2004) (‘Difference’), and positively-selected sites (‘+Sel’). Substitution matrices are substitution-matrix 1 (horizontal lines), substitution-matrix 2 (stippled), substitution-matrix 3 (diagonal lines), and substitution-matrix 4 (cross-hatched). Locations are considered buried or exposed based on whether their accessible side-chain area is larger or smaller than 10% of the areas calculated by Ahmad et al. [44]. Positively-selected sites were based on the Maximum Likelihood analysis of Yang [45]; consistently less significant correlations were observed when positively-selected sites identified by Bush et al. [10] were used. B) Log2-odds representation of the propensity of various types of locations for various numbers of substitution-matrix changes. A posteriori probabilities of changes of substitution matrix over the tree are calculated for each site. (Probabilities of substitution-matrix change less than 5% are neglected.) Sites are then assigned into one of three categories of rate of change in selective pressure from the number of changes found: slow (white, 0–1 changes), medium (gray, 1–2 changes), and fast (black, >2 changes). Arrows in the plot refer to log2-odds of negative infinity.

The amino-acid preferences of the four substitution matrices representing the four categories of selective pressure are represented in Figure 2. The distribution of types of locations described by the different substitution matrices is shown in Figure 3A, while Figure 3B shows how the number of substitution matrix changes during the evolutionary process corresponds to various types of locations. In model 4, locations can change between the different substitution matrices during the evolutionary process. The average rate of change between the different substitution matrices is tabulated in Table S2.

Substitution matrices one and two, representing 34% and 46% of all locations, respectively, are the slowest changing, with relative amino-acid substitution rates (*v_k_*) of 0.28 and 0.46 respectively. (The substitution rates are normalised so that the substitution rate averaged over all sites is 1.0) The preferred set of amino acids is largely complementary between substitution matrices one and two, with substitution matrix one having an above-average hydrophobicity compared with substitution matrix two, although substitution matrix one contains an abundance of asparagine and glutamic acid while substitution matrix two has a propensity towards aromatic residues. As would be expected, the sequence changes of buried locations are preferentially described by the predominantly-hydrophobic substitution matrix one, while exposed locations not categorised as receptor-binding or in the canonical antigenic sites are preferentially described by the more hydrophilic substitution matrix two.

Substitution matrices three and four, representing 9% and 11% of the locations in the protein, change relatively rapidly, with relative substitution rates *v_k_* equal to 2.93 and 3.90. Substitution matrix three is biased towards positively charged amino acids (arginine and lysine), while substitution matrix four has a bias towards small polar amino acids. Locations associated with the antigenic response–locations in the canonical antigenic sites and locations whose identity distinguishes the various antigenic clusters [5]–are predominantly described by these matrices, and also more likely to undergo changes in selective pressure. Loop regions are preferentially describable by substitution matrix four, in contrast to exposed coils. Receptor-binding sites are also more likely to correspond to these rapidly-evolving substitution matrices, corresponding to the high overlap between the receptor-binding and canonical antigenic sites. It is also possible that changes in the virus to prevent antibody neutralisation might involve modulating the receptor-binding properties directly, rather than inhibiting antibody binding.

Table 1 shows the sites in the protein that undergo significant changes in selective pressure during transitions between antigenic clusters. As is shown, most of the changes occur in locations in the canonical antigenic sites, but there does not seem to be a preponderance of selective pressure changes in any particular site. Some of the locations undergoing selective pressure changes correspond to locations of the cluster-difference amino acid substitutions identified by Smith and co-workers [5] (the K156Q substitution during the WU95→SY97 transition, and H75Q during the SY97→FU02 transition), while other changes in selective pressure occur near the cluster-difference substitutions (*e.g.* location 157 near the G158E substitution during the EN72→VI75 transition). There is evidence for a change in selective pressure at location 124 during the TX77→BK79 transition, with a G124D cluster-difference substitution occurring during the subsequent BK79→SI87 transition. Many changes in selective pressure, however, occur in locations that are not directly associated with cluster-difference substitutions. Haemagglutinin must fulfil a number of functional requirements. Changes in antigenic properties might be correlated with adjustments of other properties, such as receptor binding, which could then be associated with changes in selective pressure not directly associated with the antigenic response. Finally, there might be compensatory changes due to, for instance, the effect of some substitutions on thermodynamic stability.

**Table 1:** Most significant changes in selective pressure. Locations with significant probability of a given substitution-matrix change are tabulated.

	2→4	4→2	3→4	4→3	1→2
HK68→EN72		*275(C)*			**229(D)**
EN72→VI75,TX77	**164**				**157(B)**
TX77→BK79	124(A)		*138(A)*		
	*214(D)*				
BK79→SI87	**135(A)**				
SI87→BE89	**78(E)**				
SI87→BE92	**226(D)**				
BE92→WU95	**275(C)**		**92(E)**		
WU95→SY97			**156(B)**		**233**
SY97→FU02	25			*92(E)*	222
	**75(E)**				
	**225**				

The magnitude of the probability of a change are given as 0.3 to 0.4 (italics), 0.4 to 0.5 (normal), and 0.5 and above (bold). Because it is difficult to separate the transitions from EN72 to VI75 and TX77, the transition between these three states are considered a single transition. Antigenic region (where appropriate) is indicated.

The locations of different substitution matrices and changes in substitution matrix, compared with the canonical antigenic sites and those determined as being under positive selection are illustrated in Figures S2 and S3, respectively. We observe rapid substitutions, as well as rapid changes of substitution matrix, in the central exposed ‘pore’ at the top of the protein. These locations are not identified either as being under positive selection, or as changing during changes in antigenic property, although they are centrally-located and surrounded by such locations. Changes involved in cluster transitions BE92→WU95 and WU95→SY97 are shown in Figures S4 and S5, respectively.

## Discussion

There is evidence of changes in selective pressure during HA evolution. For instance, Wolf et al. recently observed transient ‘adaptive bursts’ characterised by positive selection occurring in epitopic regions [9]. In between these bursts there is little evidence for positive selection, and newly-emergent lineages only slowly replace existent lineages. There has also been evidence for non-transient shifts of selective pressure. For instance, while changes in the 18 locations identified by Bush *et al.* as under positive selection from 1983 to 1997 [10] seemed to be correlated with the subsequent phylogenetic trajectories [11] and changes in antigenic properties [5] during this same time period, changes in these 18 locations over a longer time-range were only weakly correlated with changes in antigenic properties [5],[12]. A study of sequence change and sequence variability suggests that antigenic drift involves changes in a local region, but that the location of this region varied from transition to transition [13]. All of this suggests that positive selection is a feature of influenza evolution, but that the *locations* undergoing positive selection may change and new antigenic sites may emerge.

As described in the introduction, the punctuated nature of the antigenic changes can be explained if different locations had different impacts on antigenic properties, and cluster-changes corresponded to changes at the more critical locations. In this case, the selective pressure might still be relatively constant or change in a way not correlated with the changes in antigenic properties. Alternatively, jumps in antigenic properties might represent change in the mechanism of immune-avoidance for the virus or changes in the antibody response. This latter alternative has been recently simulated with an epidemiological model [6]. The majority of substitutions occur within a cluster as populations evolve within a set of sequences with similar antigenic properties. These changes progress until a single or set of (rare) mutations cause a jump to a new antigenic variant with higher fitness. This sequence and its descendents replace the old cluster, resulting in the collapse of the population to a new single lineage that undergoes a new cycle of diversification. Our model provides evidence for the second explanation, that the antigenic clusters correspond to changing relationships in the ‘arms-race’ between influenza and the immune system, resulting in significant changes in selective pressure at different locations in the protein. These changes in selective pressure are quite rapid, corresponding to the amount of selective pressure change that would occur in a branch of length 0.7, while the branch lengths for the transitions are on the order of 0.01 to 0.03: this represents a 20- to 70-times increase in the rate of changes of selective pressure.

Consistent with this model, the changes in selective pressure occur predominantly in the canonical antigenic sites. These changes also occur at locations occupied by different amino acids in the different antigenic clusters [5]. It is important to note that these are not necessarily *cluster-defining* changes, in that some of these changes might have occurred independently of any changes in antigenic properties. Still, there is a strong correlation between sites undergoing such amino acid changes between antigenic clusters and locations where there are corresponding changes in selective pressure. There is also a significant tendency for changes in selective pressure in the regions surrounding the cluster-difference changes.

Interestingly, we cannot detect a significant increase in the rate of substitution matrix change during changes in glycosylation. As is clear from Figures S1 and 1, we also observe no correlation between changes in glycosylation and changes in antigenic clusters. *None* of the cluster-changing transitions involve a change in glycosylation site; conversely, many single antigenic clusters contain different HA with a wide range of different glycosylation states. This result is surprising, given the experimental evidence that glycosylation can reduce antibody binding [2], [14]–[17], although it is important to note that significant changes in antigenic properties can occur within the antigenic clusters.

A similar analysis of glycosylation changes in H9 evolution in birds, thought to represent a situation of viral ‘stasis’ in a natural host, do not demonstrate any significant increase in glycosylation state (data not shown). Similarly, the glycosylation state of H1 in humans does not show a substantial increase, with the number of glycosylation sites fluctuating between about 8 and 10 per subunit (data not shown). The amount of glycosylation may represent a balance between antibody shielding and other requirements such as the need to modulate receptor affinity [18]–[20] and avoid the innate immune response; increased haemagglutinin glycosylation results in *reduced* virulence in mice due to virus binding by collagenous lectins [21]. Reduced binding of influenza viruses by this mechanism in humans might alter the balance towards increased glycosylation. Another possible explanation is that glycosylation-induced antigenic changes that might occur in humans would not be detected in ferrets, and thus do not show up in the antigenic property analysis of Smith et al. It is known, for instance, that humans contain a significant number of antibodies for galactose compared with ferrets [22]. It is not clear how this would explain the absence of correlation between glycosylation changes and changes in selective pressure.

We note that we are examining changes in the predicted, rather than observed, glycosylation state. It is likely that a large fraction of these locations are, in fact glycosylated. The crystal structure of haemagglutinin from H3N2 A/Aichi/2/1968 (PDB designation 5HMG) is predicted to have six glycosylation sites per subunit, four of which are observed in the structure [23]; the remaining two might have been lost through protein expression, purification, or crystallisation. Furthermore, we consider it unlikely that the inaccuracy of the predictions is responsible for the lack of correlation between antigenic changes and glycosylation changes, as there is no reason to believe that there are significant numbers of predicted glycosylation sites that change their occupancy during changes in antigenic cluster while there are no sites that change their predicted state. Similarly, it is difficult to imagine that there is a correlation between undetected changes in occupancy of these predicted sites that correspond to increased changes in selective pressure when no such correlation is observed with changes in the predicted sites.

We find strong support for a model where the selective pressure changes preferentially during transitions between antigenic clusters. This suggests that evolution of human H3 consists of periods of amino acid variation according to a relatively constant set of rules, interspersed with periods where the rules governing variation change. These issues have important consequences for the *predictability* of antigenic drift. If the selective pressure at different locations in the protein are relatively constant, we could directly extrapolate future changes from past changes, an assumption explicit in previous analyses [11]. If, however, changes in antigenic properties are associated with changes in virus-immune system interactions, we might have to model changes in this relationship in order to perform reasonable extrapolations, as important sequence changes during one interval of antigenic drift might not be the same as ones that are important during other intervals. In addition to modelling how amino acids change during time, we also may need to develop models for how the selective pressure changes. These results also suggest that the notion of canonical ‘antigenic sites’ might be overly simplistic. It appears that there are a wide range of different locations with different propensities towards antibody recognition, and that the specific haemagglutinin locations so targeted may change with time. If so, the distinction between antigenic and non-antigenic sites may be subtle and time-dependent.

## Methods

### Evolutionary models

As described above, we develop a series of increasingly complex models. Each increase in complexity, if justified by the data, demonstrates a simplifying assumption that can be rejected, providing increased understanding of the nature of the evolutionary process in influenza.

Early simple evolutionary models, that assume that the rate of substitutions at all locations in all proteins at all times followed the same substitution matrix, have been gradually supplemented by mixture models that allow differences in the absolute substitution rates [24], relative substitution rates at different locations [25]–[28], and differences in the substitution rates at different times [29],[30]. Each component of the mixture model, represented by a distinct substitution matrix, reflects a different degree or form of selective pressure. In the simplest models (such as Gamma-distributed rate classes), we can consider different components as having different magnitudes of selective pressure, resulting in different absolute substitution rates. In the mixture models considered here, we allow for differences in the magnitude of the selective pressure as well as differences in the preferences for the different types of locations for the various amino acids. For instance, one component may model the inside of the protein, and so have a bias towards hydrophobic amino acids.

Details of the various models are described in Protocol S1. (For an overview of standard approaches to evolutionary modelling, see *e.g.* [31].) Model 1 involves a standard single substitution matrix with Gamma-distributed rate variation [24]. In Model 2, we consider that different locations in the protein follow, or ‘are assigned’, to one of a number of different possible substitution matrices [25]–[28]. We do not initially know which sites belong to which substitution matrix. Instead, each substitution matrix *k* has a specified *a priori* probability *P*(*k*) of representing any given site in the protein at any time. (As all sites must belong to some substitution matrix, 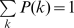). The different substitution matrices are characterised by an overall substitution rate ν*_k_*, the relative frequencies for the twenty diverse amino acids {π*_i,k_*}, and a symmetric rate parameter matrix *S_i,j_* (*S_i,j_*_ =_ *S_j,i_*) that is optimised over the entire dataset and is the same for all substitution matrices. The overall substitution rates are normalised so 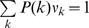. Model 3 includes a rate at which a substitution matrix describing any given location can change to another during the evolutionary time, representing variations in the selective pressure on the protein over time. The various parameters for the substitution matrix model without changes in selective pressure are {*S_ij_*, *P*(*k*), ν*_k_*, π*_i,k_*}. Allowing changes in substitution matrix adds {*Z_kl_*}, a new symmetric matrix (*Z_k,l_* = *Z_l,k_*) adding an additional *N_k_* (*N_k_*−1)/2 parameters for *N_k_* substitution matrices.

Models 4 and 5 consider the possibility that the rate of change of selective pressure, that is, the rate at which a single location changes from one substitution matrix to another, might depend upon the specific branch of the tree, depending, for instance, according to whether that branch involved a change in antigenic properties (Model 4) or glycosylation state (Model 5). In these cases, we consider a model where these specific branches are subject to an additional substitution-model change matrix which only includes substitution matrix change but no additional changes of amino acid. We can then use the likelihood ratio test to see if the resulting improvement in the log likelihood justifies the addition of this additional parameter.

### Data and adjustment of model parameters

To evaluate the models we have used the dataset of Smith *et. al.* [5] which contains 254 Human H3 HA_1_ sequences sampled from 1968 to 2003. An avian H3 sequence (A/Duck/Hokkaido/33/80, M16739) was used as an outgroup to root the tree. Sequences were extracted from the Influenza Sequence Database [32]. The Maximum Likelihood phylogenetic tree was derived using PHYML [33] with the WAG substitution model [34] and a Gamma-distributed rate [35]. Various parts of the tree were assigned to different antigenic clusters according to the designations of Smith et al [5]. The listings of these antigenic clusters as well as the abbreviations used in the text are in the legend for Figure 1. After the computation of the phylogenetic tree, the parameters of the model were optimised to maximise the log-likelihood, using software available from the authors.

The probability of the different substitution matrices and amino acids at each location in the protein for each ancestral state were calculated using standard maximum-likelihood ancestral reconstruction methods [36],[37], as were the probability of changes in selection pressure.

Ancestral glycosylation states were determined by searching for locations containing the sequence Asn-Xaa-Ser/Thr with probability >0.95. Homology models of a representative set of ML ancestral sequences were made with SwissModel [38] based on the 1MQN structure [39]. When glycosylation states were predicted by the GlyProt server [40], all potential locations were predicted to be glycosylated.

### Model choice

We are often confronted with the choice of one model or another, of varying degrees of complexity and resulting fit to the sequence data. The relative fit of two different models is quantified by the ratio of their likelihoods (that is, the probability that the observed data would be generated by the model), or equivalently, the magnitude of the change in log-likelihood. In some cases, these models are ‘nested’, that is, one model (A) is a restricted form of model (B), in which case we can use the likelihood ratio test to see if the added complexity is justified by the resulting increase in log-likelihood [41]. We cannot use the likelihood ratio test to evaluate the performance of non-nested models. Instead, we use the Akaike Information Criterion (AIC) [42], which is defined as AIC = 2*N_p_*−2Λ, where *N_p_* is the number of adjustable parameters and Λ is the log-likelihood. The preferred model is that which minimises the resulting AIC. According to this criterion, a more complex model is only justified when it causes an increase in the log likelihood greater than the number of additional parameters.

## Supporting Information

Table S1Rate of change of substitution matrix. Average rates of substitution-matrix change are represented, given by 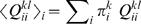.(59 KB DOC)

Table S2 Glycosylated locations for the various antigenic clusters. + refers to locations that are glycosylated in all ancestral nodes in the cluster, while # indicates a location that is glycosylated in some fraction of the nodes.
(39 KB DOC)

Figure S1 Characteristics Figure 1: Phylogenetic tree of influenza H3 HA1 sequences. Regions of the tree are colour-coded and labelled according to their antigenic cluster, as defined in (Smith et al. 2004a); labels represent the location (Hong Kong (HK), England (EN), Victoria (VI), Texas (TX), Bangkok (BK), Sichuan (SI), Beijing (BE), Wuhan (WU), Sydney (SY), Fujian (FU)) and year of the first identification. Changes in glycosylation are represented by red lines. Note that no changes of antigenic cluster correspond to changes in glycosylation.
(1763 KB TIF)

Figure S2 The mean substitution matrix (a) and number of substitution-matrix changes (b) are shown on top and side projections of a filled-sphere representation of the HA structure 1MQN [1]. Left) The mean posterior distribution of substitution matrices for each node in the tree is used to find the assignment of substitution matrix for each location. Substitution-matrix assignment is indicated by amino-acid color. The first two (slowest) substitution matrices are shown in white. Substitution-matrix three is given in green, and four in red. Where assignment is indeterminate (i.e. the posterior probabilities are spread between the substitution matrices) this is indicated by mixing of the appropriate colors. The unmodeled HA_2_ chain is shown in grey. Bold black lines around an amino acid indicate that the location is present in one of the five canonical antigenic sites. Right)The total number of substitution-matrix changes is calculated for each location over the entire tree and is shown by coloring each amino acid an appropriate shade of red: from white (no changes) to dark red (many changes). Black lines around an amino acid indicate that the location is antigenic.
(15204 KB TIF)

Figure S3 The mean substitution matrix (Left) and number of substitution-matrix changes (Right) are shown on 1MQN. Color schemes are as Figure S3. Black lines indicate that a site is predicted to be positively selected [2]. The positively selected sites were determined from a dataset published in 1999, however removal of all nodes from the tree after 1998 has a negligible effect on the mean substitution-matrix or mean number of substitution-matrix changes.
(17045 KB TIF)

Figure S4 Left) The substitution-matrix assignments for the node before the TX77→BK79 transition are plotted on the HA structure 1MQN. Colors are as Figure S3. Centre) We calculate the probability of each possible change in substitution matrix for each site along the branch corresponding to the TX77→BK79. These are indicated on the 1MQN structure by mixing the appropriate colors. Green and red indicate changes towards substitution matrices three and four respectively. Blue indicates a change towards substitution matrices one and two. White sites are unchanging. Intensity of color indicates the magnitude of the change, and colors are mixed if more than one change is taking place. Amino acids labeled with black edges are those designated as cluster-difference mutations for TX77→BK79 by Smith *et al.* [3]. Right) The substitution matrix assignments for the node after the TX77→BK79 transition. Colors are as Figure 4.
(13815 KB TIF)

Figure S5 Left) The substitution-matrix assignments for the node before the WU95→SY97 transition are plotted on the HA structure 1MQN. Colors are as Figure S3. Center) Transitions between substitution matrices along the WU95->SY97 transition are marked in color as in Figure S-5(Center). Right) The substitution-matrix assignments for the node after the WU95→SY97 transition. Amino acids labeled with black edges are those designated as cluster-difference mutations for WU95->SY97 by Smith *et al*. [3].
(12925 KB TIF)

Protocol S1 Methods: Evolutionary models
(52 KB DOC)
