# Supplementary material for: Changing Selective Pressure during Antigenic Changes in Human Influenza H3
Source: PLoS Pathog. 2008 May 2;4(5):e1000058. doi: 10.1371/journal.ppat.1000058 (PMC2323114; doi:10.1371/journal.ppat.1000058)
Supplement: Table S1 — Rate of change of substitution matrix. Average rates of substitution-matrix change are represented, given by . (59 KB DOC) [file ppat.1000058.s001.doc]

|  | **8** | **22** | **38** | **45** | **63** | **81** | **122** | **126** | **133** | **144** | **165** | **246** | **276** | **285** |
| --- | --- | --- | --- | --- | --- | --- | --- | --- | --- | --- | --- | --- | --- | --- |
| HK68 | # | + | + |  | # | # |  |  |  |  | + |  |  | + |
| EN72 | + | + | + |  | # | # |  | # |  |  | + |  |  | + |
| VI75 | + | + | + |  | + |  |  | + |  |  | + |  |  | + |
| TX77 | + | + | + |  | + |  |  | + |  |  | + |  |  | + |
| BK79 | + | + | + |  | + |  | # | + |  |  | + | # |  | + |
| SI87 | + | + | + | # | + |  |  | + |  |  | + | + |  | + |
| BE89 | + | + | + | # | + |  |  | + |  |  | + | + |  | + |
| BE92 | + | + | + |  | + |  |  | + |  |  | + | + | # | + |
| WU95 | + | + | + |  | + |  | # | # | # |  | + | + |  | + |
| SY97 | + | + | + |  | + |  | + | + | + | # | + | + |  | + |
| FU02 | + | + | + |  | + |  | + | + | + | + | + | + |  | + |
