# Supplementary material for: Changing Selective Pressure during Antigenic Changes in Human Influenza H3
Source: PLoS Pathog. 2008 May 2;4(5):e1000058. doi: 10.1371/journal.ppat.1000058 (PMC2323114; doi:10.1371/journal.ppat.1000058)
Supplement: Table S2 — Glycosylated locations for the various antigenic clusters. + refers to locations that are glycosylated in all ancestral nodes in the cluster, while # indicates a location that is glycosylated in some fraction of the nodes. (39 KB DOC) [file ppat.1000058.s002.doc]

|  | **1** | **2** | **3** | **4** |
| --- | --- | --- | --- | --- |
| **1** |  | 0.033 | 0.0 | 0.044 |
| **2** | 0.044 |  | 0.0 | 0.003 |
| **3** | 0.0 | 0.0 |  | 0.011 |
| **4** | 0.012 | 0.001 | 0.009 |  |
