# Supplementary figures and images for: Changing Selective Pressure during Antigenic Changes in Human Influenza H3
Source: PLoS Pathog. 2008 May 2;4(5):e1000058. doi: 10.1371/journal.ppat.1000058 (PMC2323114; doi:10.1371/journal.ppat.1000058)

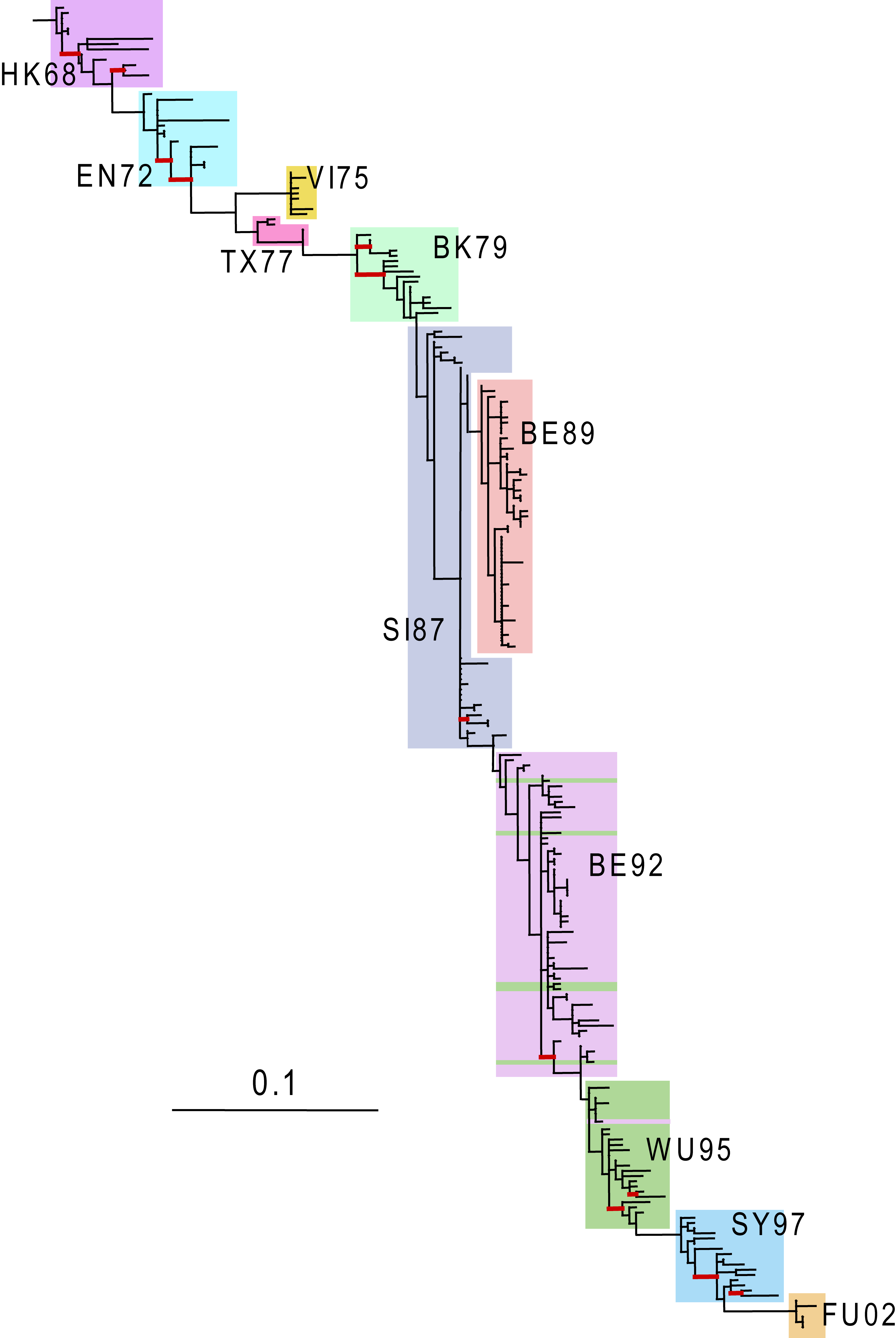

Supplement: Figure S1 — Characteristics Figure 1: Phylogenetic tree of influenza H3 HA1 sequences. Regions of the tree are colour-coded and labelled according to their antigenic cluster, as defined in (Smith et al. 2004a); labels represent the location (Hong Kong (HK), England (EN), Victoria (VI), Texas (TX), Bangkok (BK), Sichuan (SI), Beijing (BE), Wuhan (WU), Sydney (SY), Fujian (FU)) and year of the first identification. Changes in glycosylation are represented by red lines. Note that no changes of antigenic cluster correspond to changes in glycosylation. (1763 KB TIF) [file ppat.1000058.s003.tif]

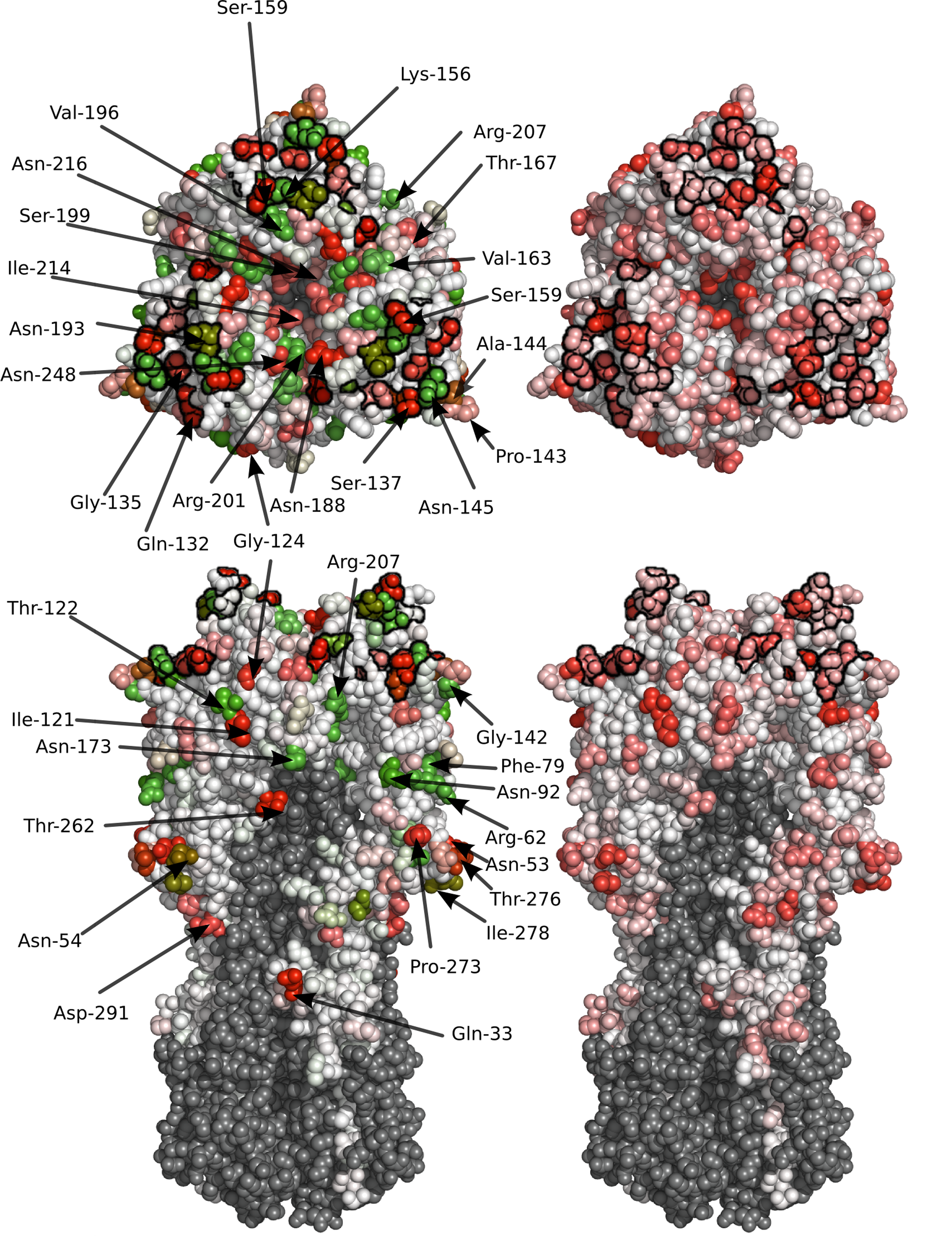

Supplement: Figure S2 — The mean substitution matrix (a) and number of substitution-matrix changes (b) are shown on top and side projections of a filled-sphere representation of the HA structure 1MQN [1]. Left) The mean posterior distribution of substitution matrices for each node in the tree is used to find the assignment of substitution matrix for each location. Substitution-matrix assignment is indicated by amino-acid color. The first two (slowest) substitution matrices are shown in white. Substitution-matrix three is given in green, and four in red. Where assignment is indeterminate (i.e. the posterior probabilities are spread between the substitution matrices) this is indicated by mixing of the appropriate colors. The unmodeled HA2 chain is shown in grey. Bold black lines around an amino acid indicate that the location is present in one of the five canonical antigenic sites. Right)The total number of substitution-matrix changes is calculated for each location over the entire tree and is shown by coloring each amino acid an appropriate shade of red: from white (no changes) to dark red (many changes). Black lines around an amino acid indicate that the location is antigenic. (15204 KB TIF) [file ppat.1000058.s004.tif]

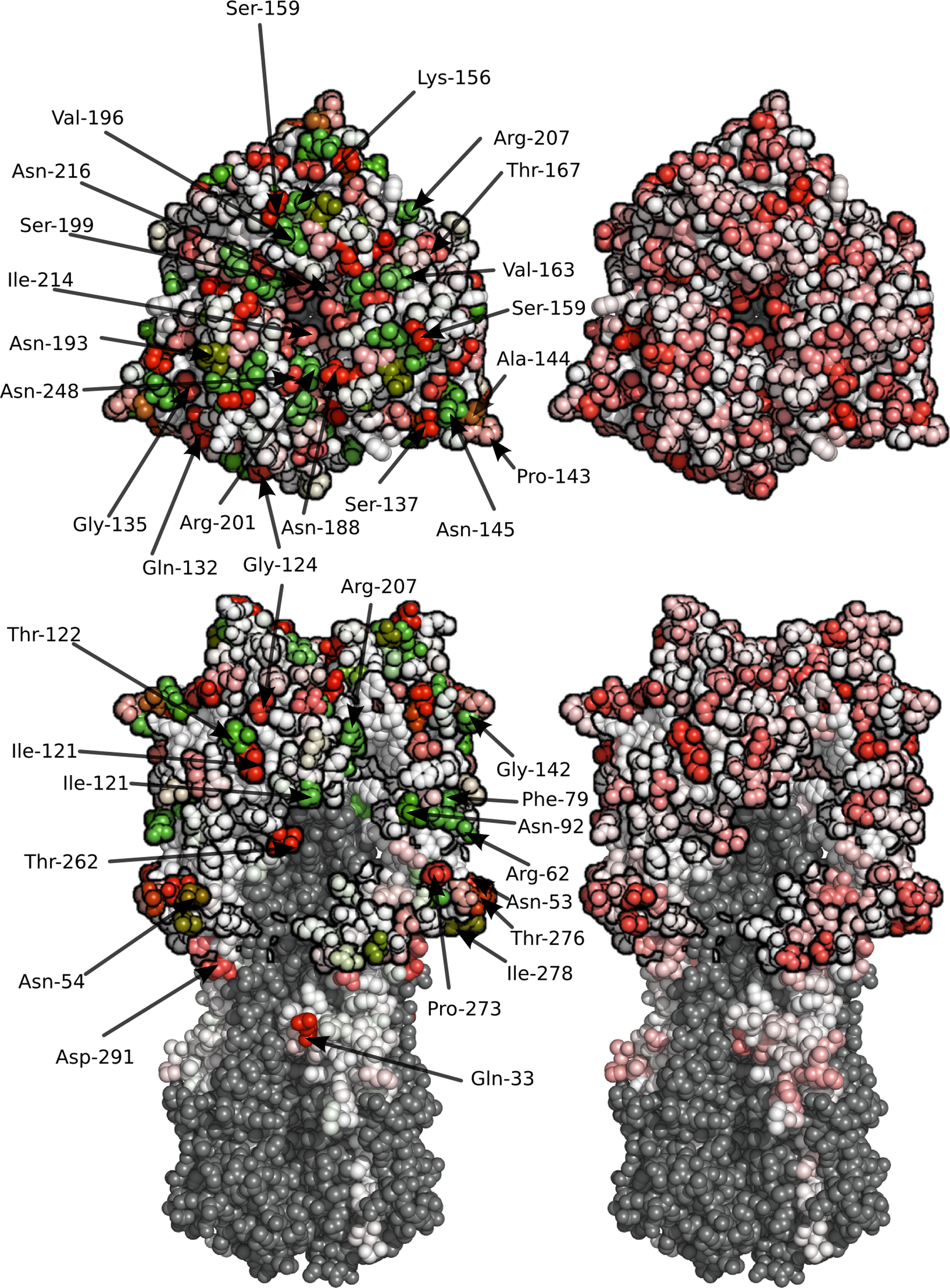

Supplement: Figure S3 — The mean substitution matrix (Left) and number of substitution-matrix changes (Right) are shown on 1MQN. Color schemes are as Figure S3. Black lines indicate that a site is predicted to be positively selected [2]. The positively selected sites were determined from a dataset published in 1999, however removal of all nodes from the tree after 1998 has a negligible effect on the mean substitution-matrix or mean number of substitution-matrix changes. (17045 KB TIF) [file ppat.1000058.s005.tif]

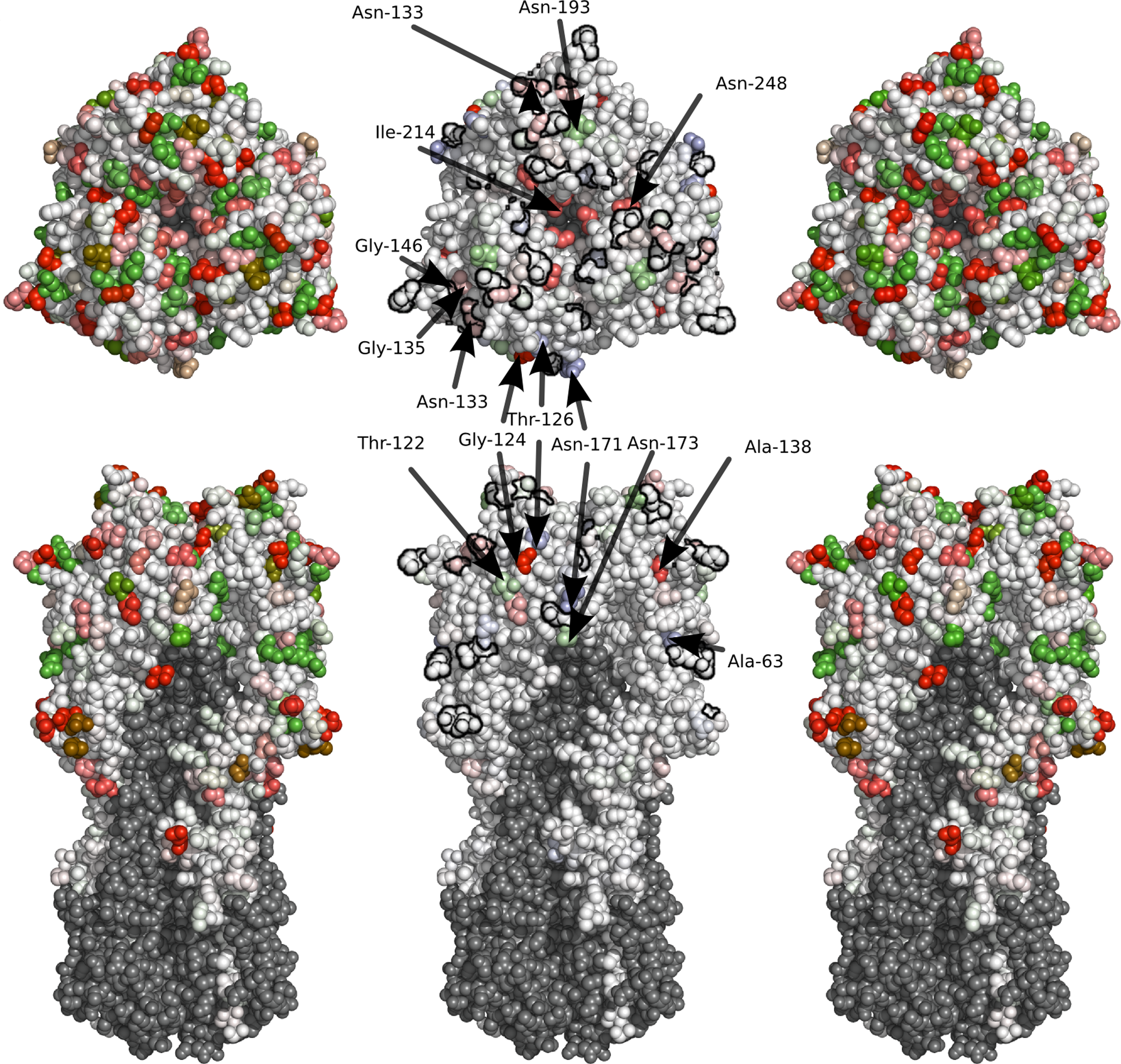

Supplement: Figure S4 — Left) The substitution-matrix assignments for the node before the TX77→BK79 transition are plotted on the HA structure 1MQN. Colors are as Figure S3. Centre) We calculate the probability of each possible change in substitution matrix for each site along the branch corresponding to the TX77→BK79. These are indicated on the 1MQN structure by mixing the appropriate colors. Green and red indicate changes towards substitution matrices three and four respectively. Blue indicates a change towards substitution matrices one and two. White sites are unchanging. Intensity of color indicates the magnitude of the change, and colors are mixed if more than one change is taking place. Amino acids labeled with black edges are those designated as cluster-difference mutations for TX77→BK79 by Smith et al. [3]. Right) The substitution matrix assignments for the node after the TX77→BK79 transition. Colors are as Figure 4. (13815 KB TIF) [file ppat.1000058.s006.tif]

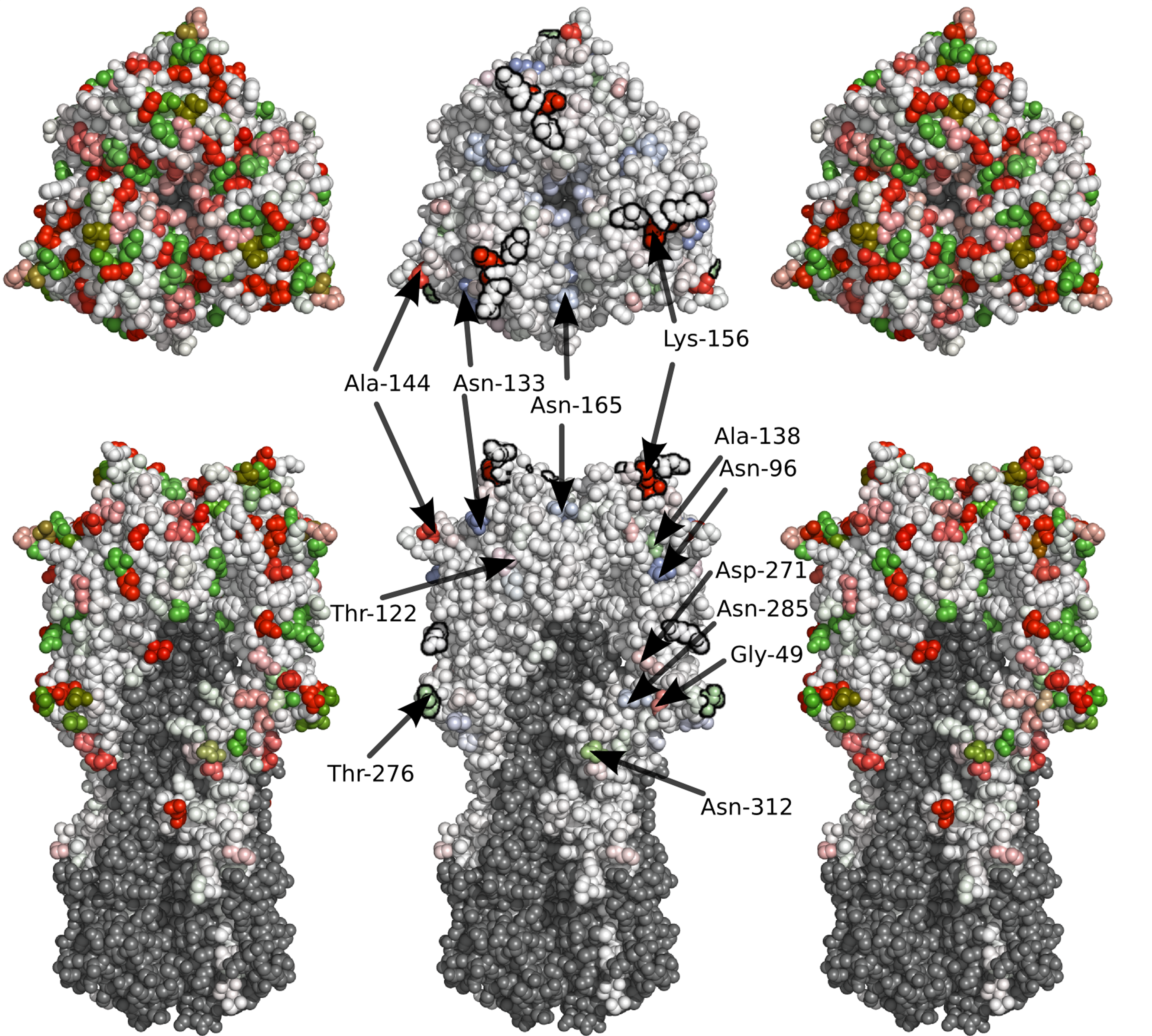

Supplement: Figure S5 — Left) The substitution-matrix assignments for the node before the WU95→SY97 transition are plotted on the HA structure 1MQN. Colors are as Figure S3. Center) Transitions between substitution matrices along the WU95->SY97 transition are marked in color as in Figure S-5(Center). Right) The substitution-matrix assignments for the node after the WU95→SY97 transition. Amino acids labeled with black edges are those designated as cluster-difference mutations for WU95->SY97 by Smith et al. [3]. (12925 KB TIF) [file ppat.1000058.s007.tif]
