# Supplementary material for: Changing Selective Pressure during Antigenic Changes in Human Influenza H3
Source: PLoS Pathog. 2008 May 2;4(5):e1000058. doi: 10.1371/journal.ppat.1000058 (PMC2323114; doi:10.1371/journal.ppat.1000058)
Supplement: Protocol S1 — Methods: Evolutionary models (52 KB DOC) [file ppat.1000058.s008.doc]

**Methods: Evolutionary models**

Early simple evolutionary models assumed that the rate of substitutions at all locations in all proteins at all times followed the same substitution matrix. These simple models have been gradually supplemented by mixture models that allow differences in the absolute substitution rates at different locations [24], differences in the relative substitution rates at different locations [25-28], and differences in the substitution rates at different times [29,30]. Each component of the mixture model, represented by a distinct substitution matrix, reflects a different degree or form of selective pressure. In the most simple models (such as Gamma-distributed rate classes), we can consider different components as having different magnitudes of selective pressure, resulting in different absolute substitution rates. In the mixture models considered here, we allow for differences in the magnitude of the selective pressure as well as differences in the preferences for the different types of locations for the various amino acids. For instance, one component may model the inside of the protein, and so have a bias towards hydrophobic amino acids.

Model 1 involves a standard single substitution matrix with Gamma-distributed rate variation [24]. In Model 2, we consider that different locations in the protein follow, or ‘are assigned’, to one of a number of different possible substitution matrices [25-28]. We do not initially know which sites belong to which substitution matrix. Instead, each substitution matrix *k* has a specified *a priori* probability *P*(*k*) of representing any given site in the protein at any time. (As all sites must belong to some substitution matrix, $\sum_{k} P(k)=1$). The different substitution matrices are characterized by an overall substitution rate ν*_k_* and the relative frequencies for the twenty diverse amino acids {π*_i,k_*}, allowing for differences in both the quantitative and qualitative nature of the substitution process. Within a substitution matrix *k*, the rate of amino acid change $Q_{i,j}^{k,k}$between *A_i_* and *A_j_* is given by

$Q_{i,j}^{k,k}=C\nu_{k}\sqrt{\frac{\pi_{j,k}}{\pi_{i,k}}} S_{i,j}$ (*i* ≠ *j*) (1)

where the symmetric matrix *S_i,j_* (*S_i,j_* = *S_j,i_*) is optimized over the entire dataset and is the same for all substitution matrices. *C* is a normalization constant chosen so that$\sum_{k} P\left(k\right) \nu_{k}=1$.

Model 3 includes a rate at which a substitution matrix describing any given location can change to another during the evolutionary time, representing variations in the selective pressure on the protein over time. We make the standard simplifying assumption of no simultaneous changes in both amino acid and substitution matrix [29,30]. $Q_{i,i}^{k,l}$ , the rate of change from substitution matrix *k* to substitution matrix *l* given that the amino acid at this location remains *A_i_*, is given by

$Q_{i,i}^{k,l}=\frac{\pi_{i,l}}{\sqrt{P(k)P(l)} \left(\sum_{i'} \pi_{i',k}\pi_{i',l}\right)} Z_{k,l}$ (*k* ≠ *l*) (2)

where *Z_k,l_* is a new symmetric matrix (*Z_k,l_* = *Z_l,k_*) representing an additional *N_k_* (*N_k_* -1)/2 parameters for *N_k_* substitution matrices. $Q_{i,i}^{k,k}$are calculated as $Q_{i,i}^{k,k}=-\sum_{j\neq i} Q_{i,j}^{k,k}-\sum_{l\neq k} Q_{i,i}^{k,l}$. For a branch of length *b*, the matrix of probabilities ***P*** for changes of substitution matrix and amino acid is given by ***P*** = exp(*b****Q***). (For an overview of standard approaches to evolutionary modelling, see *e.g.* [31].) The various parameters for the substitution matrix model without changes in selective pressure are {*S_ij_*, *P*(*k*), ν*_k_*, π*_i,k_*}. Allowing changes in substitution matrix adds {*Z_kl_*}.

Models 4 and 5 consider the possibility that the rate of change of selective pressure, that is, the rate at which a single location changes from one substitution matrix to another, might depend upon the specific branch of the tree, depending, for instance, according to whether that branch involved a change in antigenic properties (Model 4) or glycosylation state (Model 5). In these cases, we consider a model where these specific branches are subject to an additional selective-pressure change matrix which only includes substitution matrix change but no additional changes of amino acid. The probabilities of substitution matrix change are the same as for the standard matrix integrated over an effective branch-length γ: ***P*** = exp(*b****Q***) exp(γ***Q_V_***), where ***Q_V_*** is a modified form of the ***Q*** matrix so that $Q_{i,j}^{k,k}$is set equal to 0 for all *i*, *j*, and *k*. We can then use the likelihood ratio test to see if the resulting improvement in the log likelihood justifies the addition of this additional parameter.
